# The Impact of Lake Ecosystems on Mineral Concentrations in Tissues of Nile Tilapia (*Oreochromis niloticus* L.)

**DOI:** 10.3390/ani11041000

**Published:** 2021-04-02

**Authors:** Tokuma Negisho Bayissa, Sangi Gobena, Donna Vanhauteghem, Gijs Du Laing, Mulugeta Wakjira Kabeta, Geert Paul Jules Janssens

**Affiliations:** 1 Department of Nutrition, Genetics, and Ethology, Faculty of Veterinary Medicine, Ghent University, Heidestraat 19, 9820 Merelbeke, Belgium; donna.vanhauteghem@ugent.be; 2 Department of Biology, College of Natural Sciences, Jimma University, Jimma P.O. Box 378, Ethiopia; sagnigobena12@gmail.com (S.G.); enku2005@yahoo.com (M.W.K.); 3 Department of Green Chemistry and Technology, Faculty of Bioscience Engineering, Ghent University, Coupure Links 653, 9000 Gent, Belgium; gijs.dulaing@ugent.be

**Keywords:** minerals, toxic trace elements, lake ecosystems, *Oreochromis niloticus*, fillet

## Abstract

**Simple Summary:**

Fish are a source of minerals that is highly favored by consumers in most parts of the world. However, these minerals become toxic upon high-level intake and can accumulate toxic trace elements in different tissues. Nevertheless, mineral distribution in fish tissues is poorly evaluated. Analyzing tissue mineral distribution would help us to understand the physiological role of each tissue and the impact of the ecosystem on mineral and toxic trace element accumulation in the tissues. We evaluated the differences in mineral and toxic trace element concentrations of Nile tilapia (*Oreochromis niloticus*) tissues from three aquatic ecosystems. Distinct differences were observed between tissues in Nile tilapia; in addition, these concentrations were substantially affected by the lake the fish were caught from. The accumulation of elements toxic to humans, such as aluminum, should be monitored and, in particular, controlled when rearing these fish in aquaculture. Further investigation is warranted to identify the origin of the very high intestinal Fe concentration in all fish samples, which coincided with high concentrations of Al.

**Abstract:**

This study evaluates the differences in mineral and toxic trace element concentrations of Nile tilapia (*Oreochromis niloticus*) tissues from three aquatic ecosystems in Ethiopia—Lake Ziway, Lake Langano, and Gilgel Gibe reservoir—with a focus on edible (fillet) and discarded (digestive tract, gills, skin, and liver) parts. A total of sixty (*n* = 60) Nile tilapia samples were collected, comprising twenty (*n* = 20) fish from each lake, and analyzed by inductively coupled plasma mass spectrometry. All elements varied markedly among tissues and between the lakes. Some differences in element concentrations were attributed to differences in nutrient load in the ecosystems and the function of the tissues. For instance, the calcium concentrations in skin and gill were distinctly higher in fish from calcium-rich Lake Langano. The d iscarded parts were richer in essential trace elements, showing an opportunity to promote their use in human nutrition to increase the intake of important minerals. However, the accumulation of elements toxic to humans, such as aluminum, should be monitored and, in particular, controlled when rearing these fish in aquaculture.

## 1. Introduction

Food insecurity and malnutrition remain a great problem worldwide, with a prevalent burden on developing countries [1,2]. Hidden hunger critically affects human health when diet fails to meet nutrient requirements [3]. Fish are a source of minerals that is highly favored by consumers in most parts of the world [4]. Moreover, eating fish provides polyunsaturated fatty acids (PUFAs) that help reduce the risk of cancer and cardiovascular diseases [5,6,7]. Fish can, thus, play a role in fighting malnutrition and the hidden hunger problem [3,8]. Fish are also generally considered a valuable source of macrominerals, e.g., calcium (Ca), phosphorus (P), sodium (Na), magnesium (Mg), sulfur (S), potassium (K), and essential microminerals such as iron (Fe), zinc (Zn), manganese (Mn), and copper (Cu) [9]. However, essential microminerals become toxic upon high-level intake, and fish can accumulate toxic trace elements such as lead (Pb), chromium (Cr), cadmium (Cd), cobalt (Co), and nickel (Ni) [10]. Nevertheless, mineral distribution in fish tissues is poorly evaluated since studies have mainly focused on the protein and fat composition of fish fillets [11,12].

There are various ways in which fish acquire minerals [13]: by direct ingestion of suspended particulate matter in the water column in the form of food, by ion exchange of dissolved elements across lipophilic membranes (e.g., the gills), and by adsorption of elements on tissue and membrane surfaces. The elemental distribution in different tissues can be governed by way of dietary and/or aqueous exposure [13]. Analyzing different fish tissues for mineral distribution can be used to locate mineral sequestration sites, hence providing a basis for advice on the consumption of fish parts. 

In natural conditions, the distribution of minerals can vary with the local activity (farming, industrial, or urban activity) that limits the resealing rate of effluents into the nearby aquatic environment. Due to numerous coenzyme functions, minerals play an important role in human health, growth and development, and disease prevention [14]. Studies on mineral accumulation in fish are essential for understanding the effects associated with the consumption of fish by humans [15]. However, the role of fish as a dietary source of minerals is poorly recognized and underevaluated [16]. 

Nile tilapia is a common and well-accepted fish under both capture and culture conditions, especially in tropical and subtropical regions (Asia, Africa, and the Americas) [17]. Currently, it is the second most important cultured fish in the world, next to carp species. In Ethiopia, it is widely distributed in lakes, rivers, reservoirs, and swamps and contributes to about 60% of total landings of fish [18,19]. Moreover, it has the ability to consume a wide variety of food items, including phytoplankton, zooplankton detritus, and macrophytes [20,21]. The high degree of plasticity and opportunism in their feeding behavior induces variation in nutrient composition among localities; hence, different ecosystems lead to variations in diet composition. 

A report demonstrated that the nutrient composition of freshwater fish differs between geographical localities [22]. From an ecological point of view, a fair amount of research was done on how ecosystems affect fish, but the effect on the nutrient composition of fish has been overlooked. It is, however, likely that the accumulation and distribution of beneficial and toxic trace elements in fish will be especially affected by the mineral load in its environment and diet, which, in turn, will be a reflection of soil composition and geological events, such as soil erosion [23,24]. Moreover, there is limited information on the mineral concentration of discarded fish tissues (gill, skin, digestive tract, and liver), but former work has demonstrated that micromineral concentrations can show large concentration differences between tissues [25]. Because of differences in metabolic activities in each tissue and the environmental conditions, some tissues, such as the liver, accumulate more toxic trace elements that may harm consumers. 

In particular, mineral distribution within the tissues of Nile tilapia is underexplored. Analyzing tissue mineral distribution would help us to understand the physiological role of each tissue and the impact of the ecosystem on mineral and toxic trace element accumulation in the tissues of Nile tilapia. Therefore, to identify the impact of ecosystems on mineral and toxic trace element concentrations in the tissues of Nile tilapia, we evaluated the differences in mineral (Ca, P, S, Mg, Na, K, Fe, Cu, Zn, and Mn) and toxic trace element (Cr, Ni, Pb, Cd, and Co) concentrations of Nile tilapia from three aquatic ecosystems—Lake Ziway (LZ), Lake Langano (LL), and Gilgel Gibe reservoir (GR)—with a focus on edible (fillet) and nonedible or discarded parts (digestive tract, gills, skin, and liver). The catchment areas of the water sources are different for the three lakes. In recent days, commercial floriculture and water pump irrigation by local farmers have rapidly expanded on the shoreline of Lake Ziway and along its tributary rivers [26]. The lake is also under heavy pollution pressure because of expanding urbanization: Batu and Maki are the fast-growing towns near Lake Ziway [26]. Compared to other lake basins, Lake Langano experiences only small seasonal water level variations. However, in the surrounding lake, there is an increasing number of resorts and tourists. As a result, untreated effluents are directly discharged into the lake; this could have negative effects [22]. The water in Gilgel Gibe Reservoir is collected from three agricultural streams (Merewa, Gibe, and Gulufa), three urban streams (Kito, Kochi, and Awetu), and one forest stream. These streams pass through fast-growing urban and intensive agricultural areas. Untreated wastewater, generated by Jimma town inhabitants, is directly discharged into the urban stream, which is the main tributary of the reservoir. Municipal waste discharge, overgrazing, brick preparation, vegetation removal and land conversion to cropland, drainage, and crop cultivation are the major threats from human activities around the tributaries of the reservoir [27]. 

## 2. Materials and Methods

### 2.1. Description of the Study Area

Fish samples (Nile Tilapia) from LZ, LL, and GR were used in this study, and a summary of limnological information is given in Table 1 [18,26,27]. Lake Ziway is located in the central Rift Valley zone of Ethiopia. It has open water and flat swampy margins on all sides except the south and southwest. It fills a depression at an elevation of about 1636 m above sea level. Meki and Ketar Rivers are the main tributaries of the lake. The lake is the shallowest lake in the country and drains into Lake Abiyata via Bulbula River. It is the third-largest freshwater lake in the country. It has a surface area of 434 km^2^ and has five islands: Gelila, Debre Sina, Tulu Gudo, Tsedecha, and Fundro. The climate of the lake basin is dry to subhumid. The lowland area surrounding the lake is arid or semiarid, and the highlands are subdry humid to humid. Lake Langano is one of the northern Rift Valley lakes of Ethiopia. It is situated at an altitude of 1582 m and has a surface area of 241 km^2^ and a mean depth of 17 m. The lake is highly turbid, and the water is usually reddish-brown; it is known by its nickname “Golden Lake”. It is mainly fed by runoff and hot springs. The lake water discharges into Lake Abijata via Hora Kello River. The climate is rainy between June and September, followed by a dry season from November to February. Gilgel Gibe reservoir is located 283 km southwest of the capital and 70 km northeast of Jimma town. The dam is constructed at an altitude of 1640 m above sea level. Its maximum and minimum water levels during wet and dry seasons are 1671 and 1653 m above sea level, respectively. Its depth ranges between 2 and 35 m, with a mean depth of about 17.6 m, and it covers a total surface area of 62 km^2^. It is bordered by three districts (woredas): Omo-Nada, Kersa, and Tiro-Afeta. The area has a subhumid, warm to hot climate and receives between 1300 and 1800 mm of annual rainfall; it has a mean annual temperature of 23.7 °C. In addition to hydropower generation, the reservoir plays an important role for the local community as a fishery resource.

### 2.2. Water Quality Parameters of the Three Lakes

On-site physicochemical water quality measurements of the three lakes were performed. Day-time dissolved oxygen, water temperature, electrical conductivity, and pH were measured using a multiprobe meter (HQ40d Single-Input Multi-parameter Digital Meter; Hach Company, Loveland, CO, USA). In addition, the concentration of nitrate and ammonia were also measured on-site using a Palin test photometer (Photometer 7500; Tyne and Wear, UK, NE11 0NS ) ( Table 2). 

### 2.3. Fish Sample Collection and Preparation 

Nile tilapia (*O. niloticus*) samples were collected from LZ, LL, and GR during the dry season (October 2018). Nile tilapia was selected based on its relevance for commercial fishing in the country and fish consumption by the local population. The target tissues were chosen based on the edible and discarded parts. A total of sixty (*n* = 60) fish samples were collected, comprising 20 (*n* = 20) fish from each lake, caught using standard gillnets. Fish samples were thoroughly washed with tap water, separately wrapped in plastic foil (parafilm), placed on ice, and brought to the laboratory. Fish from Gilgel Gibe Reservoir were transported to Jimma University Zoological Sciences, whereas fish from Lake Ziway and Lake Langano were brought to the Batu Fisheries and Other Living Aquatic Resource Research Center. Upon arrival, total body weight and total body length of all fish were measured to the nearest 0.1 g and 0.01 cm, respectively. Because of the too-small sample amount of tissues such as the liver, the fish samples were randomly pooled into three groups (6, 6, and 7 fish). Each pooled sample was analyzed in triplicate. All materials used in the handling and dissecting of the fish samples were washed in distilled water in advance. Plastic tools (knife and forceps) were used to collect the target tissues, and the samples were stored at −20 °C until further analysis. The digestive tract was analyzed with its contents to have an idea about the mineral intake of the fish. The skin was analyzed with scales, and, in this study, both skin + scales are referred to as “skin”; this is because of an assumption that scales can potentially be a rich source of minerals. The concentrations of macrominerals, microminerals, toxic trace elements, and ash content were determined in all targeted tissues.

### 2.4. Percentage of Ash Determination

Three grams of the dried samples were placed in a preweighed crucible. Before placing in the oven, the samples in the crucible cups were charred on hot plates for 30 min to prevent excess smoke production. For ashing, the weighed samples were incinerated in a muffle furnace (550 °C) for 6 h, and the percentage of ash content was calculated [28]. 

### 2.5. Mineral Analysis

The fillet and discarded tissue parts of the fish samples were packed and shipped to Ghent University, Belgium, for the analysis of minerals and toxic trace elements. Following the methodology described by Association of Official Analytical Chemists, AOAC (1990), the samples were weighed on a microbalance (Mettler Toledo, AT21 comparator), homogenized, and allowed to react for 12 h in a mixture of 3 mL HNO_3_ and 3 mL H_2_O_2_. Subsequently, the samples were heated in a microwave oven (CEM, MARS6) with the following program: 10 min at 55 °C and 400 W, followed by 10 min at 75 °C and 600 W, and, finally, 40 min at 120 °C and 1200 W. The extracts were analyzed by inductively coupled plasma mass spectrometry (ICP-MS; PerkinElmer, Elan DRCe) with a calibration range of 0–50 mg/L. The instrument of detection (LOD) for ICP-MS was calculated as the concentration associated with 3.3 times the standard deviation of the background noise recorded on 9 measurements of the procedural blank and given in online resources. Extracts were diluted 1:10 (*v*/*v*) with Ga as an internal standard solution. The signal of the sample analyte was always within the standard curve, and the ISO 11885 international standard method was used to calibrate the instrument. The quantification limits of the analyzed elements were set at 0.005 mg/L for Al, Cr, Cu, Fe, Zn, and Mn, 0.01 mg/L for Cd and P, 0.05 mg/L for Co, Mg, Na, S, Ca, and K, 0.1 mg/L for Ni, and 0.2 mg/L for Pb.

### 2.6. Nutritional Contribution Assessment 

Possible mineral contributions in a 100-g serving of Nile tilapia tissues from the three Ethiopian lakes to a percentage of the daily value of foods were simulated based on a dry-matter basis of the samples. For the nutritional contribution assessment, the calculated intake of the analyzed essential elements per 100 g serving of Nile tilapia tissues was compared to the dietary reference intake (Recommended Daily Allowance and Adequate Index) for women aged between 19 and 30 years as an example that is set by the Food and Nutrition Board, Institute of Medicine, United States National Academy of Sciences [29,30,31]. 

### 2.7. Statistical Analysis

All data were displayed on a scatterplot to check for normality. The effects of tissues on mineral, heavy metal, and ash concentrations were evaluated with a general linear model—repeated measure analysis of variance (ANOVA). One-way analysis of variance (ANOVA) was also used to determine the effect of locality on mineral and toxic trace element concentrations. For both analyses, a subsequent posthoc comparison using Tukey’s test was performed. All analyses were done using the statistical package of SPSS version 26.0. Significance was evaluated at *p* < 0.05 confidence level.

## 3. Results

Across the lakes, the concentrations of macrominerals Na, Mg, and K were higher in the digestive tract compared to other tissues. However, in the three lakes, the concentrations of Ca and P were markedly higher in the skin and gill tissues compared to other tissues. Gills from Lake Langano contained more Ca compared to the other two lakes. There was no considerable difference in muscle ash content between the lakes. The ash content in gill and skin was lowest in Gilgel Gibe fish compared to the other two lakes. However, higher ash content was observed in the digestive tract and liver from Lake Langano fish compared to the other two lakes (Table 3).

The concentration of the evaluated microminerals (Fe, Zn, Cu, and Mn in mg/kg dry matter) varied greatly between the tissues and the lakes (Table 4). A remarkably higher concentration of Fe was observed in the gill tissue from Lake Ziway and Gilgel Gibe fish than Lake Langano fish. However, Fe concentration in the liver tissue from Gilgel Gibe fish was higher than from the other two lakes. There was no significant variation in Zn concentration in digestive tract tissue between the three lakes, but it varied in gill and skin. Mn concentration was higher in the skin and digestive tract from Gilgel Gibe fish. Muscle from Lake Langano had a distinctly higher Cu concentration compared to the other two lakes. However, the liver samples from the same lake showed lower Zn concentrations than the two other lakes.

The measured concentrations of toxic trace elements (Al, Cr, Co, Ni, and Cd in mg/kg dry matter) varied depending on the analyzed tissue and the origin of the sample (Table 4). The highest concentrations were found in liver and digestive tract tissues in the three lakes. For instance, the liver and the digestive tract from Gilgel Gibe fish contained higher concentrations of cobalt than the same tissues from the other two lakes, but tissue cobalt concentrations did not vary between lakes. Muscle, gill, and skin contained lower concentrations of Al and Cr than in digestive tract and liver tissues across the three lakes. The concentration of cadmium was below the detection limit in all analyzed tissues and lakes, except in the liver tissue from Lake Ziway. Digestive tract samples from Gilgel Gibe and Ziway contained notably higher concentrations of Al than other tissues, but these were several-fold lower than samples from Lake Langano. 

The percentage contribution of Nile tilapia tissues from the three Ethiopian lakes per 100 g serving to a mineral’s dietary recommended intake (DRI) is summarized in Table 5 for women aged between 19 and 30, as an example. Gill and skin provided a higher percentage of DRI of Ca and liver provided a higher percentage of DRI for Fe, Na, and Cu than other tissues per 100 g of serving for women. 

Mineral concentrations of Nile tilapia tissues in the present study were also compared with United States Department of Agriculture (USDA) composition data for raw Tilapia fillets, as presented in Table 6. All Nile tilapia tissues, particularly gill, skin, and liver, had pronounced higher concentrations of all minerals than USDA composition data.

## 4. Discussion

Ash is the indication of overall mineral content in the samples [32]. From the ash and individual element concentrations, it is clear that muscle (fillet) is not a rich source of minerals. Therefore, encouraging the consumption of other tissues, or the entire fish, could substantially increase mineral intake in the human diet.

Higher ash content in the digestive tract likely reflects high mineral concentrations in the diet, potentially including sediment. In several commercially important fish species [22,33], ash content responded to changes in localities. The sum of all analyzed elements only represents a fraction of the total ash in the digestive tract, suggesting that silicon-containing soil dominates total ash content in the digestive tract samples. The higher ash concentration in LZ digestive tract may, therefore, indicate the eutrophic status of the lake and a more particulate-suspension-feeding strategy of the fish in that lake [26], resulting in a high intake of insoluble, nonabsorbable minerals, as can be seen from the lower ash concentration in the liver of LZ fish, as well as the high intestinal Ca:P ratio, typical for calcium-based soil material. 

The “bony” structures in gills and skin can explain the higher deposition of Ca and P in those tissues. Both external tissues deposit hydroxyapatite in the vitrodentin of the scales, composed of collagen covered with Ca [34]. In particular, in these tissues, the highest concentrations of these minerals were observed in fish from LL, showing an adaptation of these fish to the high electric conductivity of this lake. Likely the high Na concentrations in gills and skin in the same lake adhere to that principle as well. This means that the environment affects macromineral concentrations in fish tissues. 

Lake Langano is indeed a lake with high Na and Ca concentrations in the water [35] compared to the two other lakes. In contrast to gills and skin, the low Ca concentration in the digestive tract of LL fish means that diet items are not necessarily higher in Ca in contrast to the water. However, reports have indicated that the elevated concentration of minerals in gills reflects a higher concentration in the diet [36], which may still hold for some of the microminerals, as will be discussed further. 

Most S in the body is found in S-containing amino acids such as methionine and cysteine. The latter is especially needed to make strong protein structures through disulfide bonds. It is, therefore, logical to find the highest concentrations, again, in skin and gills and fairly low concentrations in muscle, despite its high protein content. Here, too, the high S concentration in LL skin and gills corresponds with the apparent need for stronger structures in the gills and skin to resist the high conductivity and mineral load in that lake. The high S concentrations in the digestive tract —and especially those from GG—are, again, likely to reflect the high S concentrations in the diet items because of the importance of disulfide bonds in the strength of chitin [37], a structure dominating the exoskeleton of zooplankton [38].

Further investigation is warranted to identify the origin of the very high intestinal Fe concentration in all fish. Since it coincides with high concentrations of Al, the intake of suspended soil or the accidental intake of particulate matter during suspension feeding may be a plausible explanation. The three lakes are surrounded by Al- and Fe-rich soils [26,27,39], increasingly challenged by soil erosion [40]. Although Fe is an essential mineral for animals, extremely high concentrations may become a burden to the fish; although Fe uptake is strongly regulated, hepatic accumulation seems to occur [41,42]. Fish are known to regulate elevated Fe concentrations in the whole body by controlling the level in blood, subsequently transferring Fe to the liver for storage [43]. As LL has more Ca-rich soils, the problem is less apparent than in LZ and GG. The overall picture regarding Fe confirms the report by [44] that essential minerals can be found in fish tissues, with the highest concentrations in the liver, gills, kidneys, and spleen and the lowest concentrations in skin and muscle. The liver has a crucial function in basic metabolism, (exchangeable) mineral storage, redistribution, and detoxification or transformation [45,46]. 

Iron deficiency causes anemia, and fish are a major source of Fe [47]. In the present study, the Fe concentration of the liver, digestive tract, and gills of Nile tilapia is several-folds higher than in muscle and skin. Therefore, the liver and gills of Nile tilapia may be a good source of iron for human nutrition, depending on dietary needs. Similarly, Cu, Zn, and Mn were also higher in those tissues compared with muscle. 

Fish liver and gills accumulate not only essential elements like Fe, Cu, Zn, and Mn, which may also become toxic at too-high uptake levels but also toxic trace elements like Cr, Al, Cd, Ni, and Co (Table A1) [10]. It is, therefore, important to be careful in the consumption of Nile tilapia liver due to the high content of these toxic trace elements. For this reason, only the liver and gills of Nile tilapia that are harvested or cultured under controlled environmental conditions can be considered safe for consumption.

According to the US Food and Drug Administration [48], food that provides more than 10% of nutrients per serving is considered a good source of that nutrient. In this study, mineral contribution to DRI per 100-g serving was calculated (Table 5). Nile tilapia tissues from the three Ethiopian lakes of this study can provide more than 10% of minerals (Ca, P, Fe, Zn, Cu, Mg, Mn, Na, K) for women aged between 19 and 30, hence making Nile tilapia tissues (other than muscle) good sources of these minerals. This comparison is made to show that small inclusions of such parts (tissues) in the diet may substantially improve the intake of certain minerals. Moreover, a comparison of mineral concentrations in tilapia tissues from the three Ethiopia lakes to USDA composition data for raw tilapia showed that Nile tilapia tissues from the three lakes had different concentrations of minerals compared to USDA composition data [48] (Table 6). This possibly indicates that wild tilapia may have higher mineral concentrations than fish in cultured conditions, and this should be considered in the contribution of fish to human mineral intakes. Several factors could contribute to these differences, such as diet, geographical variations, the environment that the fish were grown in, and the methods that were used to measure the minerals.

The concentration of heavy metals in water influences the content of toxic trace elements in fish tissues(Table A3) [49]. In our study, the highest concentrations of these toxic trace elements were recorded in the liver and digestive tract, followed by gills, and the lowest in muscle and skin. This agrees with other reports that the metal accumulation capacity of muscles in fish is lower than that of other tissues such as gills, gut, and liver (Table A2) [50]. Cadmium is a nonessential element found in natural waters, with potential toxicity to fish even at low levels [13]. The detection of Cd only in the liver tissue from LZ could be related to intensive human activities carried out in every corner of that lake. A recent publication reported that the lake is under increasing agricultural and urban pressure and is exhibiting deteriorating trends in several water-quality and ecological parameters; additionally, toxic trace element concentrations of the lake have shown increasing temporal trends [39]. Similarly, the higher Al accumulation in the digestive tract from Gilgel Gibe and Ziway fish is likely caused by the high inflow of eroded topsoil due to intensive unprotected farming activities in the surrounding ecosystems. Whereas the other toxic trace elements were still at acceptably low concentrations (Table A1), the high concentrations of Al in the digestive tract may be a concern for human consumption as it exceeds the maximum tolerable intake [51]. 

## 5. Conclusions

As a conclusion, distinct differences in minerals and toxic trace element concentrations were observed between tissues in Nile tilapia. In addition, these concentrations were substantially affected by the lake these fish were caught from. Some differences could be attributed to the water quality of the lakes, whereas others may refer to different food webs. Compared to fillet (muscle), the typically noneaten parts were much richer in minerals, leaving an opportunity to fortify human nutrition with essential minerals. However, caution is needed due to the accumulation of some toxic trace minerals such as Al, likely arising from anthropogenic soil erosion. Moreover, the route by which these toxic trace elements enter into fish tissue should be optimally identified, and responsible bodies should pay attention to public health safety. Quality control for toxic trace elements in water and diet is therefore warranted when culturing these fish.

## Figures and Tables

**Table 1:** Geographic description of the three Ethiopian lakes in this study.

Parameters	GG	LZ	LL
Latitude	7°42′53″–7°55′580″ N	7°20′54″–8°25′56″ N	7°03′0″–7°04′2″ N
Longitude	37°11′53″–37°20′33″ E	38°13′02″–9°24′01″ E	38°04′0″–38°04′9″ E
Altitude (m) asl	1640	1636	1582
Surface area (km^2^)	62	434	241
Catchment area (km^2^)	4200	7025	1600
Mean depth (m)	17.6	2.5	17
Distance from the capital	283 km, southwest	165 km, south	200 km, south

Cited from [18,26,27]. GR: Gilgel Gibe reservoir; LZ: Lake Ziway; LL: Lake Langano.

**Table 2:** Physicochemical water quality parameters of the three Ethiopian lakes in this study.

Lake	T (°C)	DO (mg/L)	EC (μS/cm)	pH	NH_4_^+^ (mg/L)	NO_3_^−^ (mg/L)
Ziway	26.98	6.31	1345	8.55	0.64	7.97
Langano	27.41	6.11	1634	8.73	0.68	9.26
G/Gibe	23.29	6.74	456	7.56	0. 71	2.81

(In situ measurement); T = temperature, DO = dissolved oxygen, EC = electric conductivity.

**Table 3:** The concentration of ash and macrominerals in Nile tilapia tissues from three lakes in Ethiopia (g/kg dry matter; pooled samples (*n* = 3 from each lake)).

Element/Ash	Tissues	GR	LZ	LL	*P*	SEM
Ash	Muscle	59	63	69	0.190	4.7
	Gill	153 ^a^	199 ^b^	205 ^b^	<0.001	9.5
	Skin	135 ^a^	226 ^b^	234 ^b^	<0.001	7.1
	Digestive tract	323 ^a^	371 ^b^	316 ^a^	0.001	10.6
	Liver	143 ^b^	121 ^a^	139.9 ^b^	0.010	6.9
Ca	Muscle	2.2	2.3	2.4	0.803	0.3
	Gill	49 ^a^	66 ^ab^	76 ^b^	0.007	4
	Skin	46 ^a^	58 ^a^	95 ^b^	<0.001	3
	Digestive tract	13	17	9	0.186	3
	Liver	3	2	5	0.768	2
Na	Muscle	1	3	2.3	0.059	0.4
	Gill	4.9 ^a^	6.7 ^b^	8.9 ^c^	0.013	0.3
	Skin	1.3 ^a^	1.1 ^a^	6.1 ^b^	0.024	0.9
	Digestive tract	26	12	25	0.089	4
	Liver	12	13	11	0.782	2
Mg	Muscle	1.4	0.9	0.8	0.054	0.1
	Gill	1.2 ^b^	1.5 ^c^	0.7 ^a^	<0.001	0.1
	Skin	1.2 ^b^	1.1 ^b^	0.5 ^a^	<0.001	0.1
	Digestive tract	3.9 ^b^	4.8 ^b^	1.8 ^a^	<0.001	0.2
	Liver	1.7 ^b^	1 ^a^	0.6 ^a^	0.043	0.1
S	Muscle	0.1	0.1	0.2	0.238	0.05
	Gill	0.7 ^a^	0.2 ^a^	1.7 ^b^	0.002	0.1
	Skin	0.1 ^a^	0.2 ^a^	1.3 ^b^	<0.001	0.03
	Digestive tract	1.5 ^b^	0.5 ^a^	0.6 ^a^	0.003	0.1
	Liver	0.1	0.4	0.3	0.064	0.06
P	Muscle	10	8	8	0.258	1
	Gill	27 ^a^	37 ^b^	44 ^b^	0.003	2
	Skin	26 ^a^	31 ^a^	50 ^b^	<0.001	2
	Digestive tract	24 ^b^	9 ^a^	14 ^a^	0.018	2
	Liver	25 ^b^	15 ^a^	13 ^a^	0.003	1
K	Muscle	18	14	15	0.379	2
	Gill	5	6	7	0.132	1
	Skin	6.0 ^b^	3.5 ^a^	4.8 ^ab^	0.013	0.4
	Digestive tract	28 ^b^	15 ^a^	17 ^ab^	0.047	3
	Liver	26 ^b^	17 ^a^	14 ^a^	0.008	2

GR: Gilgel Gibe reservoir; LZ: Lake Ziway; LL: Lake Langano. Different superscripts within a row indicate significant differences at *p* < 0.05.

**Table 4:** Concentrations of trace elements in Nile tilapia tissue from three lakes in Ethiopia (mg/kg dry matter; pooled samples (*n* = 3 from each lake)).

Element	Tissues	GR	LZ	LL	P	SEM
Mn	Muscle	3	1	14	0.432	7
	Gill	5	5	3	0.223	1
	Skin	10 ^b^	5 ^ab^	4 ^a^	0.036	1
	Digestive tract	1.5 ^b^	0.4 ^a^	0.5 ^a^	0.001	0.1
	Liver	2.6	2.4	1.3	0.305	0.4
Fe	Muscle	228	249	237	0.451	21
	Gill	482	537	547	0.711	57
	Skin	53	97	115	0.168	21
	Digestive tract	16,196 ^b^	15,405 ^b^	5573 ^a^	0.004	1389
	Liver	2497 ^b^	783 ^a^	621 ^a^	0.002	208
Zn	Muscle	15	24	47	0.084	8
	Gill	52 ^a^	76 ^b^	75 ^b^	0.025	5
	Skin	55 ^a^	63 ^a^	93 ^b^	0.014	7
	Digestive tract	178	124	141	0.083	14
	Liver	178	95	128	0.083	22
Cu	Muscle	2.1^a^	1.6^a^	5^b^	<0.001	0.2
	Gill	2	3	6	0.269	2
	Skin	1.2	1.6	4.3	0.119	0.9
	Digestive tract	30	37	29	0.683	7
	Liver	1816 ^b^	1099 ^b^	126^a^	0.049	406
Al	Muscle	111	259	105	0.335	41
	Gill	113	124	193	0.430	40
	Skin	29	64	216	0.253	74
	Digestive tract	18,406 ^b^	23,604 ^b^	9411 ^a^	0.017	983
	Liver	461	702	366	0.694	203
Cr	Muscle	0.80	0.93	0.96	0.364	0.08
	Gill	1.47	1.88	1.29	0.251	0.23
	Skin	2.04 ^c^	1.41 ^b^	0.75^a^	0.001	0.12
	Digestive tract	11.7 ^a^	17.3 ^b^	12.6^a^	0.024	0.9
	Liver	15.6 ^b^	2.3 ^ab^	1.3^a^	0.047	3.2
Co	Muscle	<0.1	<0.1	<0.1	-	-
	Gill	<0.1	<0.1	<0.1	-	-
	Skin	<0.1	<0.1	<0.1	-	-
	Digestive tract	<0.1	<0.1	<0.1	-	-
	Liver	11 ^b^	7 ^a^	3 ^a^	<0.001	0.5
Ni	Muscle	<0.1	<0.1	<0.1	-	-
	Gill	<0.1	<0.1	<0.1	-	-
	Skin	<0.1	<0.1	<0.1	-	-
	Digestive tract	7 ^b^	2 ^a^	2 ^a^	0.011	1.4
	Liver	5.3 ^b^	1.3 ^a^	1.3 ^a^	0.001	1.3
Cd	Muscle	<0.01	<0.01	<0.01	-	-
	Gill	<0.01	<0.01	<0.01	-	-
	Skin	<0.01	<0.01	<0.01	-	-
	Digestive tract	22	26	8	0.167	1.3
	Liver	<0.01	0.07	<0.01	-	-
	Muscle	<0.2	<0.2	<0.2	<0.2	-
Pb	Gill	<0.2	<0.2	<0.2	<0.2	-
	Skin	<0.2	<0.2	<0.2	<0.2	-
	Digestive tract	<0.2	<0.2	<0.2	<0.2	-
	Liver	<0.2	<0.2	<0.2	<0.2	-

GR: Gilgel Gibe reservoir; LZ: Lake Ziway; LL: Lake Langano. Different superscripts within a row indicate significant differences at *p* < 0.05.

**Table 5:** Contribution (%) of Nile tilapia tissues from the three Ethiopia Lakes to dietary reference intake (DRI *) for females aged between 19 and 30, as an example (compared with USDA standard reference).

	GR				LZ				LL				Ref.
	Muscle	Gill	Skin	Liver	Muscle	Gill	Skin	Liver	Muscle	Gill	SKIN	Liver	
Mg	45	38	38	54	29	48	35	32	26	22	16	19	68
Na	3	12	3	30	7	17	3	32	6	22	15	27	23
K	69	19	23	100	53	23	13	65	57	26	18	53	64
Ca	22	490	460	30	23	660	580	20	24	760	950	50	8
P	143	386	371	357	114	529	442	214	114	629	714	186	243
Fe	126	267	29	1387	138	298	53	435	131	303	63	345	31
Zn	19	65	68	222	30	96	78	118	58	93	116	160	30
Cu	23	22	13	20177	17	33	17	12211	55	66	47	1400	83
Mn	17	28	55	14	6	28	28	13	77	16	22	7	16

GR, Gilgel Gibe reservoir; LZ, Lake Zeway; LL, Lake Langano. Ref.: reference. * Dietary reference intake (Recommended Daily Allowance) for women aged between 19 and 30 years is set by the Food and Nutrition Board, Institute of Medicine, United States National Academy of Sciences [29,30,31].

**Table 6:** Comparison of mineral concentrations (mg/100 g DW) of Nile tilapia tissues from three Ethiopian lakes with USDA compositional data for raw tilapia fillets.

Minerals	Tissues	GR	LZ	LL	USDA Composition Data
Mn	Muscle	0.3	0.1	1.4	0.37
	Gill	0.5	0.5	0.3	
	Skin	1	0.5	0.4	
	Liver	0.26	0.24	0.13	
Fe	Muscle	22.8	24.9	23.7	5.6
	Gill	48.2	53.7	54.7	
	Skin	5.3	9.7	11.5	
	Liver	249.7	78.3	62.1	
Zn	Muscle	1.5	2.4	4.7	3.3
	Gill	5.2	7.6	7.5	
	Skin	5.5	6.3	9.3	
	Liver	17.8	9.5	12.8	
Cu	Muscle	0.21	0.16	0.5	0.75
	Gill	0.2	0.3	0.6	
	Skin	0.12	0.16	0.43	
	Liver	181.6	109.9	12.6	
Ca	Muscle	220	230	240	100
	Gill	4900	6600	7600	
	Skin	4600	5800	9500	
	Liver	300	200	500	
Na	Muscle	100	300	230	520
	Gill	490	670	890	
	Skin	130	110	610	
	Liver	1200	1300	1100	
Mg	Muscle	140	90	80	270
	Gill	120	150	70	
	Skin	120	110	50	
	Liver	170	100	60	
P	Muscle	1000	800	800	1700
	Gill	2700	3700	4400	
	Skin	2600	3100	5000	
	Liver	2500	1500	1300	
K	Muscle	1800	1400	1500	3020
	Gill	500	600	700	
	Skin	600	350	480	
	Liver	2600	1700	1400	

## Data Availability

Data are available in a publicly accessible repository that does not issue DOIs. Publicly available datasets were analyzed in this study. This data can be found at https://sharepoint.ugent.be/projects/201803345/Tokuma/Forms/AllItems.aspx, accessed on 23 February 2020.

## Appendix A

**Table A1:** The tolerable values of some toxic trace elements in the fish (mg/kg).

Organization	Cd	Cr	Pb	Ni	References
IAEA-407	0.18	0.73	0.12	0.60	[52]
TKB	0.1		1.0		[53]
Directive -2005/78 EC	0.5		0.2		[54]
FAO/WHO			0.5		[55]

## Appendix B

**Table A2:** Summary of minerals and toxic elements reported by different authors from Nile tilapia tissues.

Minerals	Muscle	Gill	Liver	References
Ca mg/kg(ww)	248		350	
P mg/kg(ww)	2005		1833	
Mg mg/kg(ww)	265		93	[56]
K mg/kg(ww)	4125		1385	
Na mg/kg(ww)	353		1003	
Fe mg/kg(dw)	65	196	710	
Cu mg/kg(dw)	2.33	8.3	779	
Zn mg/kg(dw)	24.9	92	116	[57]
Mn mg/kg(dw)	1.01	35	21	
Cd mg/kg(dw)	0. 5	<0.010	1.70	
Ni mg/kg(ww)	2.39	2.32	2.68	
Cr mg/kg(ww)	1.99	1.26	0.77	[58]
Pb mg/kg(ww)	2.26	2.59	4.63	

## Appendix C

**Table A3:** Summary of the mean elemental concentrations in the water column and/or sediments from the three lakes, reported by different authors.

Elements	GR		LZ		LL	References
	Water (mg/L)	Sediment (mg/ kg DW)	Water (mg/L)	Sediment	Water (µg/L)	
Ca	2.75	-	18	-	31 mg/L	[59,60,61]
K	0.32	-	14	-	-	[59,60]
Na	0.86	-	72	-	-	[59,60]
P	2.0	-	<0.01	-	-	[59,60]
Mg	1.7	-	8.1	-	-	[59,60]
Fe	2.09	-	2. 6	1680	567.9	[59,61,62]
Cu	-	9.74	<0.01	105	1.5	[59,60,61,62]
Mn	-	-	0.033	76,900	29.9	[60,61,62]
Zn	-	-	<0.01	140	3.2	[60,61,62]
Cr	-	22.93	1.4 (µg/L)	52.3	20	[59,61,62]
Co	-	10.10	-	60.3	-	[60,61,62]
Ni	-	-	-	46.8	0.6	[59,61]
Pb	-	19.59	-	10.1	0.2	[59,61,62]
Cd	-	10.4	-	8.87	6.49 (mg/L)	[59,61,62]

GR, Gilgel Gibe reservoir; LZ, Lake Zeway; LL, Lake Langano.
